# The Derivation and External Validation of a Fibrosis Risk Model for Colorectal Tumours Undergoing Endoscopic Submucosal Dissection

**DOI:** 10.3390/jcm13154517

**Published:** 2024-08-02

**Authors:** Sandro Sferrazza, Marcello Maida, Giulio Calabrese, Antonio Facciorusso, Lorenzo Fuccio, Leonardo Frazzoni, Roberta Maselli, Alessandro Repici, Roberto Di Mitri, João Santos-Antunes

**Affiliations:** 1 Gastroenterology and Endoscopy Unit, “ARNAS Civico-Di Cristina-Benfratelli” Hospital, 90127 Palermo, Italy; giulio.calabrese@unina.it (G.C.);; 2 Department of Medicine and Surgery, University of Enna ‘Kore’, 94100 Enna, Italy; 3 Gastroenterology Unit, Ospedale Umberto I, 94100 Enna, Italy; 4 Gastroenterology Unit, Department of Medical Sciences, University of Foggia, 71122 Foggia, Italy; 5 Department of Medical and Surgical Sciences, University of Bologna, 40123 Bologna, Italy; 6 IRCCS Azienda Ospedaliero-Universitaria di Bologna, 40138 Bologna, Italy; 7 Morgagni-Pierantoni Hospital, 47121 Forli, Italy; 8 Endoscopy Unit, Humanitas Clinical and Research Hospital, IRCCS, 20089 Rozzano, Italy; 9 Department of Biomedical Sciences, Humanitas University, 20089 Pieve Emanuele, Italy; 10 Gastroenterology Department, Centro Hospitalar Universitário São João, 4200-319 Porto, Portugal

**Keywords:** ESD, fibrosis, risk, colorectal lesions

## Abstract

**Background:** Endoscopic submucosal dissection (ESD) is an advanced technique that can become more challenging in the presence of submucosal fibrosis. Predicting the grade of fibrosis is important in order to identify technically difficult ESD. **Aims and Methods:** Our study aimed to derive and validate a prediction model to determine the preoperative degree of submucosal fibrosis in colorectal tumours undergoing ESD. A predictive model was developed to derive the probability of an increasing submucosal fibrosis in the derivation cohort and then externally validated. **Results:** 309 patients (age: 68 ± 10.9 years) underwent colorectal ESD between January 2016 and June 2020. F0, F1, and F2 fibroses were reported in 196 (63.4%), 70 (22.6%), and 43 (13.9%) cases, respectively. R0 resection was found in 266 (87%) lesions. At multivariable analysis in the derivation cohort, lesion morphology (OR = 0.37 and CI = 0.14–0.97 for LST-NG vs. 0-Is; OR = 0.29 and CI = 0.1–0.87 for the LST mixed type vs. 0-Is; and OR = 0.32 and CI = 0.1–1.03 for LST-G vs. 0-Is) and increasing size (OR = 1.02 and CI = 1.01–1.04 for a 1 mm increase) were significantly associated with an increasing degree of fibrosis. The model had fair discriminating ability in the derivation group (AUROC = 0.61 and CI = 0.52–0.69 for F1–F2 vs. F0 fibroses; AUROC = 0.61 and CI = 0.45–0.77 for F2 vs. F0–F1 fibroses) and in the validation group (AUROC = 0.71 and CI = 0.59–0.83 for F1–F2 vs. F0 fibroses; AUROC = 0.65 and CI = 0.52–0.77 for F2 vs. F0–F1 fibroses). **Conclusions**: Our findings introduce a new tool for the stratification of ESD technical difficulty based on lesion size and morphological characteristics which could become crucial during the procedure’s planning process.

## 1. Introduction

Endoscopic submucosal dissection (ESD) is the preferred endoscopic treatment for the en bloc resection of large colorectal lesions with suspected submucosal invasion [1]. Unlike piecemeal snare resection, en bloc resection performed by ESD allows a more accurate histological staging along with fewer local recurrences, representing an alternative to surgery for lesions at low risk of developing lymph node metastasis.

Despite its benefits, the adoption of ESD in Western countries has been slow, and the procedure was initially proposed in Japan in the 1990s and began to be performed in European tertiary referral centres only for a decade, with less satisfactory results in efficacy and safety [2]. Notably, ESD is a challenging technique that requires highly experienced endoscopists and can involve a reasonable degree of risk. Thus far, many factors, including novice endoscopists, large tumour size, non-rectal or flexure location, and deep submucosal infiltration, have been identified as high-risk factors for unsatisfactory ESD outcomes [3,4,5,6,7,8,9,10]. Among them, submucosal fibrosis is regarded as a major feature that significantly hinders the success of ESD due to difficulties in separating the submucosal layer from the muscle one, resulting in a higher incidence of incomplete resection and perforation, as well as a longer procedure time [3,11,12,13]. Therefore, establishing the grade of fibrosis is important in order to identify technically difficult ESD that requires higher endoscopic skill and more adequate pre-operative preparation to overcome difficulties caused by submucosal fibrosis. In contrast, only a few Asian studies have reported diagnostic measures to assess the grade of submucosal fibrosis in colorectal tumours. Matsumoto et al. [14] first reported that severe fibrosis, assessed with endoscopy, was associated with the morphology of colorectal lesions; the incidence of severe fibrosis in nodular mixed-type granular lateral spreading tumours (LSTs) was significantly higher than in homogeneous-type ones [14]. Other studies have shown that protruding lesions, deep submucosal invasion, tumour size ≥ 30 mm or ≥40 mm, fold convergence, and underlying semilunar fold were also preoperative predictors for severe submucosal fibrosis during ESD [11,15,16]. Chiba et al. [16] demonstrated independent predictive factors for each grade of fibrosis, as a cecal location, non-LST granular pseudo-depression (LST-NG-PD), a previous biopsy, a straddling fold, and a protruded lesion were predictive factors of mild fibrosis and the latter two were predictive factors of severe fibrosis [16]. Recently, Cecinato et al. [17] found that left and right colonic localisation, together with non-granular morphology and invasive pit patterns, were independently associated with a higher degree of fibrosis.

However, none of these studies attempted to create a prediction tool for sub-mucosal fibrosis based on endoscopic assessment. Therefore, our study aimed to develop and validate a prediction model to determine the preoperative degree of submucosal fibrosis in colorectal tumours and, therefore, to identify technically difficult ESD.

## 2. Materials and Methods

### 2.1. Patients

This multicentre prospective study was performed at three institutions, i.e., Trento (Italy), Porto (Portugal), and Tokyo (Japan), between January 2021 and October 2022. Consecutive patients with naïve superficial colorectal lesions undergoing ESD were considered for inclusion. The following inclusion criteria had to be satisfied: (i) patient age ≥ 18 years, (ii) complete procedure reporting, and (iii) a detailed histological evaluation of the resected specimen. All recurrent and previously attempted resection or biopsied lesions were excluded. The following data were extracted for each patient: age, colorectal lesion morphology (according to the Paris and laterally spreading tumour (LST) classification) [18], as well as lesion size and site. Pit pattern was classified according to the classification proposed by Kudo [19] and the Japan NBI Expert Team (JNET) [20]. R0 resection was defined when free lateral and vertical margins were present, together with the absence of lymph vascular invasion and the presence of superficial submucosal invasion. Complications were rated according to the American Society for Gastrointestinal Endoscopy (ASGE) 2010 report [21].

### 2.2. Endoscopic Submucosal Dissection Technique

At each centre, the ESDs were performed by a single endoscopist with high experience in therapeutic endoscopy using CO_2_ insufflation and high-resolution last-generation endoscopes (GIF-HQ190, CF-HQ190I, and PCF-H190T/I (Olympus, Tokyo, Japan)). A solution of 1:100,000 diluted adrenaline and indigo carmine in a glycerol hypertonic solution (10% glycerin and 5% fructose in normal saline) was employed for submucosal lifting. A mucosal incision was performed with a 1.5 mm dual knife. Dual-knife, insulated tip (IT)-2 or IT-nano knives (Olympus©, Tokyo, Japan) were used for submucosal dissection. After dissection, hemostasis was performed with a Coagrasper© (Olympus, Tokyo, Japan) whenever necessary, and defects were closed with through-the-scope clips. An electrosurgical unit VIO3 (ERBE Elektromedizin, Tübingen, Germany) was used. We planned all ESD, according to the technique described by Prof. Yamamoto [22], and a pocket creation method was proposed as a strategy for difficult-to-resect lesions [23].

### 2.3. Outcomes

The primary outcome of the study was the presence of submucosal fibrosis, as assessed by the endoscopist during the procedure. The following grading system was applied, according to the original classification of Matsumoto A. et al. [14]: F0, no fibrosis; F1, mild fibrosis; and F2, severe fibrosis. To be more specific, F0 was defined as a transparent submucosal layer; F1 as a white web-like structure in the transparent submucosal layer; and F2 as a white muscular-like structure without a transparent submucosal layer 1. A predictive model was developed to derive the probability of increasing submucosal fibrosis in the derivation cohort (Porto and Tokyo), and then externally validated in a validation cohort (Trento). We followed the transparent reporting of a multivariable prediction model for individual prognosis or diagnosis (TRIPOD) recommendations [24]. We provided the TRIPOD checklist in Supplementary Table S1.

### 2.4. Statistical Analysis

Categorical variables were described as absolute frequencies and percentages, whereas continuous variables were reported as mean and standard deviations (SDs) or medians with an interquartile range (IQR) when not normally distributed. Comparisons between categorical and continuous variables were undertaken with the chi-square test, the unpaired *t*-test, or the Mann–Whitney U test, as well as the analysis of variance (ANOVA) as appropriate. Multivariable ordinal regression analysis was performed in the derivation cohort to identify predictive factors of increasing fibrosis. A predictive model for the risk of fibrosis was generated in the derivation cohort and then externally validated in the validation cohort. The discriminating ability of the model was assessed through the area under the receiver-operating characteristic curve (AUROC). Analyses were conducted using STATA software version 16 (Stata Corp, College Station, TX, USA).

## 3. Results

**Figure 1:**
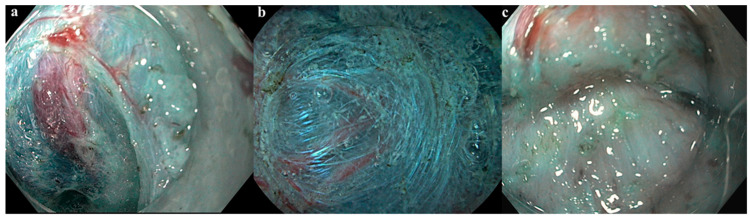
Different degrees of fibrosis graded according to Matsumoto et al. classification. (**a**). F0—no fibrosis; (**b**). F1—mild fibrosis; (**c**). F2—severe fibrosis.

Overall, 309 patients with a mean age of 68 ± 10.9 years underwent colorectal ESD between January 2016 and June 2020 and were subsequently included in the present study. Lesions were located in the rectum (33.3%), left colon (26.6%), and right colon (40.1%). The mean lesion size was 36 ± 17 mm. According to the Paris and LST classification, 10.4% of lesions were 0-Is, whereas 15.2% were LST-G-H (LST granular homogeneous), 34.6% were LST-G-M (LST granular mixed), and 39.8% were LST-NG (LST non-granular). The median procedure duration was 60 (IQR 41–95) min, and en bloc resection was achieved in 99% of cases. No fibrosis (F0), F1 fibrosis, and F2 fibrosis were reported in 196 (63.4%), 70 (22.6%), and 43 (13.9%) cases, respectively. Figure 1 shows different degrees of fibrosis. 

Curative R0 resection was achieved in 266 (87%) lesions. Complications were experienced in 20 (6.4%) resections, i.e., 10 (3.2%) being perforations and 10 (3.2%) significant bleeding. Among non-R0 resections, 19 (7.4%) patients underwent surgery due to deep submucosal invasion. Among 61 lesions followed up at 3–6 months, the recurrence rate was 0.3%. Among 96 lesions followed up at 6–18 months, no recurrence was observed (Table 1). 

The duration of ESD was significantly influenced by submucosal fibrosis, as the times were as follows: 63 + 44 min for F0, 83 + 48 min for F1, and 114 + 67 min for F2 (*p* < 0.001). Likewise, the rate of R0 resection was significantly associated with the degree of fibrosis, as demonstrated by the following percentages: 87.8% for F0, 92.9% for F1, and 67.4% for F2 (*p* < 0.001). Fibrosis was tendentially related to complication rate, as bleeding and perforation occurred in 3.1% and 2.6% of cases in F0 fibrosis, in 2.9% and 1.4% of cases in F1 fibrosis, and in 4.7% and 9.3% of cases in F2 fibrosis (*p* = 0.09). The outcomes of ESD according to submucosal fibrosis are reported in Table 2.

### Fibrosis Risk Model Derivation and Validation

The derivation cohort was composed of 199 patients enrolled in Tokyo and 32 in Porto (n = 231), whereas the validation cohort comprised 78 patients from Trento. Table 3 shows the demographic and clinical characteristics of included patients. After multivariable ordered logistic regression analysis was conducted in the derivation cohort, the following variables were significantly associated with an increasing degree of fibrosis (F1–F2 vs. F0 fibroses and F2 vs. F0–F1 fibroses): lesion morphology (OR = 0.37 and CI = 0.14–0.97 for LST-NG vs. 0-Is; OR = 0.29 and CI = 0.1–0.87 for the LST mixed type vs. 0-Is; and OR = 0.32 and CI = 0.1–1.03 for LST-G vs. 0-Is) and increasing size (OR = 1.02 and CI = 1.01–1.04 for a 1 mm increase) (see Table 4). The model had fair discriminating ability in the derivation group (AUROC = 0.61 and CI = 0.52–0.69 for F1–F2 vs. F0 fibroses; AUROC = 0.61 and CI = 0.45–0.77 for F2 vs. F0–F1 fibroses) and in the validation group (AUROC = 0.71 and CI = 0.59–0.83 for F1–F2 vs. F0 fibroses; AUROC = 0.65 and CI = 0.52–0.77 for F2 vs. F0–F1 fibroses).

**Figure 2:**
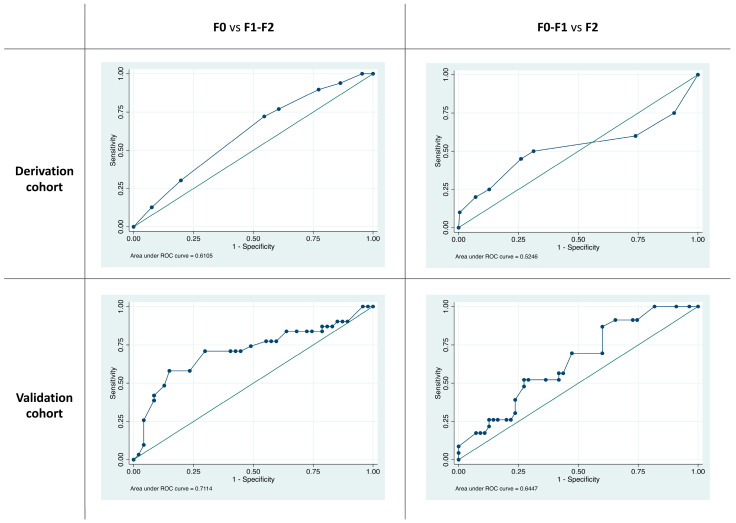
Corresponding ROC curve for F1–F2 vs. F0 fibroses in the derivation group (AUROC = 0.61 and CI = 0.52–0.69 for F1–F2 vs. F0 fibroses; AUROC = 0.61 and CI = 0.45–0.77 for F2 vs. F0–F1 fibroses) and in the validation group (AUROC = 0.71 and CI = 0.59–0.83 for F1–F2 vs. F0 fibroses; AUROC = 0.65 and CI = 0.52–0.77 for F2 vs. F0–F1 fibroses).

Corresponding ROC curves are depicted in Figure 2. Based on the model, we estimated the probability of F1–F2 and F2 fibroses, according to various lesion morphologies and sizes (see Table 5).

## 4. Discussion

In the present multicentre prospective study, which used a large sample of superficial colorectal lesions, we demonstrated that ESD outcomes, such as procedure duration, R0 resection, and complication rates, are influenced by the degree of submucosal fibrosis. Lesion morphology and increasing size were predictors of a higher degree of fibrosis following multivariable analysis. Based on these findings, we derived and externally validated a predictive model for fibrosis to pre-operatively estimate the degree of lesion fibrosis, thus allowing a more rational resource allocation for ESD. Previous studies have reported that severe fibrosis, when compared with non-severe fibrosis, resulted in prolonged procedure times, lower curative resections, and major complication rates. Severe fibrosis was associated with non-granular-type LSTs, protruding morphologies, larger tumour sizes, submucosal invasion, the presence of fold convergence, and underlying semilunar folds [11,14,15,25]. However, none of these studies assessed the ESD outcomes and predictive factors for each grade of submucosal fibrosis. Our study demonstrated that various lesion morphologies and sizes have different degrees of submucosal fibrosis (F1–F2 vs. F0 fibroses and F2 vs. F0–F1 fibroses), which results in different procedural times and incomplete resection and complication rates. As a tumour increases in size, the accompanying fibrosis is more likely to worsen. Several tumour sizes were specified to categorize severe versus non-severe fibroses. Previous studies found that tumours > 30 mm and >40 mm contained significantly more severe fibroses [11,15]. These different cut-off points could have resulted from the different mean sizes of tumours included in each study (31.8 ± 11 mm vs. 43.9 ± 19.1 mm). Our study overcomes this limitation, demonstrating a linear relationship between the risk of fibrosis and the lesion size. In particular, the risk of submucosal fibrosis, from F0 to F1 and from F1 to F2, respectively, increases by 2% for each millimetre in lesion size. The main advantage of a linear relationship between lesion size and the probability of severe fibrosis is the possibility of avoiding pre-fixed cut-offs, which are heterogeneous across the literature. Based on this finding, the probability of submucosal fibrosis, according to lesion morphology and size, was predicted. According to the results from previous studies, the evaluation of fibrosis does not seem to be affected by biases based on the assessment methods. Larger tumour sizes and lesion morphologies are indeed indifferently associated with severe fibrosis evaluated either through an endoscopist’s [15] or a pathologist’s assessment [11]. Hence, the evaluation of submucosal fibrosis performed in the current protocol is not affected by bias. Moreover, in order to minimize further possible biases, we developed our model based on naïve lesions, excluding all those lesions where a previous biopsy was performed, and resection was attempted or found to be a recurrence, which are known risk factors for moderate-to-severe fibrosis [26,27]. In our study, the invasive pit pattern was not found to be associated with a higher degree of fibrosis, unlike the study recently conducted by Cecinato et al. [17]. One possible reason that could explain this difference concerns the characteristics of the sample. In our study, 19.4% of Kudo Vi lesions were included, while in the one from Cecinato et al. [17], Vi-Vn lesions comprised 51.7% of the whole sample. Vn Kudo lesions carried a higher risk of submucosal invasion; thus, including them in the analysis may have increased the rate of severe fibrotic lesions in that sample.

Notably, the application of our predictive model in clinical practice could become useful in planning the estimated procedure time, the need for an already trained endoscopist to perform the procedure or the need for a multidisciplinary approach (including surgeons). Additionally, it could be convenient to plan and adapt the dissection technique according to the predicted degree of fibrosis, i.e., adopting a specific ESD method, as suggested by the current literature [28,29]. In a study conducted by Yoshida et al. [28], the pocket creation method could indeed guarantee a significantly higher en bloc resection rate (95.2 vs. 74.7, *p* = 0.03) and a shorter mean procedure time (79.6 ± 26.5 vs. 118.8 ± 71.0, *p* = 0.001) compared to the non-pocket creation method for LSTs with signs of severe fibrosis. These results are consistent with the previous findings by Ide D. et al. [29] where the use of the pocket creation method was associated with a traction device with clinical efficacy for residual colorectal lesions, affected by severe fibrosis. In order to overcome the problem of severe submucosal fibrosis, Morimoto S. et al. [30] recently proposed executing hybrid salvage ESD on 115 out of 1039 colorectal lesions with characteristics of technical difficulty (i.e., severe fibrosis, difficult scope position, and difficult access or need for reduced procedural time due to advent of complications). The hybrid technique guaranteed a reduced procedural time (71 min vs. 90 min, *p* = 0.0053), although the en bloc resection rate did not significantly differ between hybrid and conventional ESD (94% vs. 87%, *p* = 0.0914).

According to the results from our study, it is reasonable to state that lesions with more than 50% probability of F1 fibrosis or those with more than 20% probability of F2 fibrosis should be resected by experienced endoscopists.

The current study has several strengths; for example, its prospective design and multicentre nature with external validation make the results extremely reproducible in other tertiary referral centres. Moreover, to our knowledge, this is the first multivariable prediction model that has been derived and externally validated to pre-operatively assess the degree of submucosal fibrosis among ESD colorectal lesions. This could represent a useful decision-making tool for the endoscopist to improve resource management and allocation. Regarding the population enrolled, the characteristics of the sample (which involves a large number of participants) are homogeneous in terms of lesion site, morphology, and mucosal/vascular pattern, which allows for a low rate of bias.

The main limitation of this study is that the procedures were performed by a single endoscopist per centre, who also esteemed the degree of fibrosis on a case-by-case basis. This could represent a source of bias, which was partly reduced by the multicentre nature of the study. Moreover, it was not possible to calculate an inter-observer agreement in the grading of fibrosis, given that every lesion was evaluated by a single endoscopist.

## 5. Conclusions

Our findings introduce a new tool for the stratification of ESD technical difficulty based on lesion size and morphological characteristics, which could become crucial during the procedure’s planning process. The external validation of this model allows for its prompt use in estimating the procedural time, the need for advanced technical skills, and the necessity for surgical dissection techniques. Further studies applying this model are expected to evaluate its real-life application and its ability to predict patient outcomes, such as the risk of intra- or post-procedural complications.

## Figures and Tables

**Table 1:** Characteristics of lesions.

Variable	Study Cohort n = 309
Centre, n (%)	
Tokyo	199 (64.4)
Trento	78 (25.2)
Porto	32 (10.4)
Male sex, n (%)	122 (39.6)
Mean age, years (SD)	68.1 (10.9)
Lesion site, n (%)	
Proximal colon	124 (40.1)
Distal colon	82 (26.6)
Rectum	103 (33.3)
Lesion size, mm (SD)	36 (17.0)
Lesion morphology, n (%)	
0-Is	32 (10.4)
LST-G-H	47 (15.2)
LST-G-M	107 (34.6)
LST-NG	123 (39.8)
Kudo classification, n (%)	
II	19 (6.2)
III-L	36 (11.7)
III-S	14 (4.5)
IV	148 (47.9)
Vi	60 (19.4)
Missing	32 (10.3)
JNET classification, n (%)	
1	17 (6.2)
2A	130 (47.3)
2B	109 (39.6)
3	3 (1.1)
Missing	16 (5.8)
Median procedure duration, min (IQR)	60 (41–95)
Fibrosis, n (%)	
F0	196 (63.4)
F1	70 (22.6)
F2	43 (13.9)
Curative R0 resection, n (%)	266 (86.0)
Complications, n (%)	20 (6.4)
Perforations	10 (3.2)
Bleeding	10 (3.2)
Recurrence rate, n (%)	
3–6 months ^a^	0.3
5–18 months ^b^	0

LST: laterally spreading tumour; LST-G-H: LST granular homogeneous; LST-G-M: LST granular mixed; LST-NG: LST non-granular; F0: no fibrosis; F1: mild fibrosis; F2: severe fibrosis. ^a^ Analysis performed on 61 lesions; ^b^ analysis performed on 96 lesions.

**Table 2:** ESD outcomes according to the degree of lesion fibrosis.

Outcome	F0 (n = 196)	F1 (n = 70)	F2 (n = 43)	*p*
ESD duration, min (mean ± SD)	63 ± 44	83 ± 48	114 ± 67	<0.001
R0 resection, n (%)	172 (87.8)	65 (92.9)	29 (67.4)	<0.001
Overall complications, n (%)	11 (5.6)	3 (4.3)	6 (14)	0.092
Bleeding	6 (3.1)	2 (2.9)	2 (4.7)	0.800
Perforation	5 (2.6)	1 (1.4)	4 (9.3)	0.073

**Table 3:** Characteristics of lesions according to derivation and validation cohort.

Variable	Derivation Cohort n = 231	Validation Cohort n = 78	*p*-Value
Male sex, n (%)	97 (42)	25 (32.5)	0.139
Mean age, years (SD)	58.6 (11.1)	66.6 (10.3)	0.172
Lesion site, n (%)			<0.001
Proximal colon	120 (52)	4 (5.1)	
Distal colon	71 (30.7)	11 (14.1)	
Rectum	40 (17.3)	63 (80.7)	
Lesion morphology, n (%)			<0.001
Is	19 (8.2)	13 (16.7)	
LST-G-H	69 (29.9)	38 (48.7)	
LST-G-M	105 (45.4)	18 (23.1)	
LST-NG	38 (16.5)	9 (11.5)	
Kudo classification, n (%)			<0.001
II	19 (8.2)	0	
III-L	29 (12.6)	7 (9)	
III-S	7 (3)	7 (9)	
IV	97 (42)	51 (65.4)	
Vi	47 (20.4)	13 (16.6)	
Missing	32 (13.9)	0	
JNET classification, n (%)			<0.001
1	17 (8.5)	0	
2A	126 (63.3)	4 (5.3)	
2B	40 (20.1)	69 (90.8)	
3	0	3 (3.9)	
Missing	16 (8)	0	

**Table 4:** Multivariable model for lesion fibrosis, according to ordered logistic regression.

Variable	OR (95%CI)	*p*
Lesion morphology		
0-Is	1	-
LST-G-M	0.29 (0.1–0.87)	0.026
LST-G-H	0.32 (0.1–1.03)	0.057
LST-NG	0.37 (0.14–0.97)	0.044
Lesion increasing size (mm)	1.02 (1–1.04)	0.050

LST: laterally spreading tumour; LST-G-H: LST granular homogeneous; LST-G-M: LST granular mixed; LST-NG: LST non-granular; CI, confidence interval; LST, lateral spreading type; LST-G, granular type; LST-NG, non-granular type; OR, odds ratio.

**Table 5:** Predicted probability of lesion submucosal fibrosis based on lesion morphology and size, according to the model. Moderate-to-severe (F1–F2) fibrosis > 50% and severe (F2) fibrosis > 20% probabilities are coloured in red, and any others are coloured in green.

	0-Is		LST Mixed		LST-G		LST-NG	
	F1–F2	F2	F1–F2	F2	F1–F2	F2	F1–F2	F2
20 mm	53%	23%	24%	8%	19%	6%	32%	11%
40 mm	64%	32%	32%	11%	25%	8%	42%	16%
60 mm	74%	42%	44%	17%	35%	12%	52%	22%
80 mm	81%	53%	55%	24%	46%	18%	63%	31%

## Data Availability

Data are available upon reasonable request to the corresponding author.

## Supplementary Materials

The following supporting information can be downloaded at: https://www.mdpi.com/article/10.3390/jcm13154517/s1. Table S1: TRIPOD Checklist: Prediction Model Development and Validation.
